# Novel non-invasive biomarkers that distinguish between benign prostate hyperplasia and prostate cancer

**DOI:** 10.1186/s12885-015-1284-z

**Published:** 2015-04-11

**Authors:** Andrej Jedinak, Adam Curatolo, David Zurakowski, Simon Dillon, Manoj K Bhasin, Towia A Libermann, Roopali Roy, Monisha Sachdev, Kevin R Loughlin, Marsha A Moses

**Affiliations:** 1 Vascular Biology Program and Department of Surgery, Boston Children’s Hospital, Boston, MA USA; 2 Harvard Medical School, Boston, MA USA; 3 Department of Anesthesia, Boston Children’s Hospital, Boston, MA USA; 4 Genomics and Proteomics Center, Beth Israel Deaconess Medical Center, Boston, MA USA; 5 Department of Urology, Brigham and Women’s Hospital, Boston, MA USA

**Keywords:** Non-invasive biomarkers, Benign prostate hyperplasia, Prostate cancer

## Abstract

**Background:**

The objective of this study was to discover and to validate novel noninvasive biomarkers that distinguish between benign prostate hyperplasia (BPH) and localized prostate cancer (PCa), thereby helping to solve the diagnostic dilemma confronting clinicians who treat these patients.

**Methods:**

Quantitative iTRAQ LC/LC/MS/MS analysis was used to identify proteins that are differentially expressed in the urine of men with BPH compared with those who have localized PCa. These proteins were validated in 173 urine samples from patients diagnosed with BPH (N = 83) and PCa (N = 90). Multivariate logistic regression analysis was used to identify the predictive biomarkers.

**Results:**

Three proteins, β2M, PGA3, and MUC3 were identified by iTRAQ and validated by immunoblot analyses. Univariate analysis demonstrated significant elevations in urinary β2M (*P* < 0.001), PGA3 (*P* = 0.006), and MUC3 (*P* = 0.018) levels found in the urine of PCa patients. Multivariate logistic regression analysis revealed AUC values ranging from 0.618 for MUC3 (*P* = 0.009), 0.625 for PGA3 (*P* < 0.008), and 0.668 for β2M (*P* < 0.001). The combination of all three demonstrated an AUC of 0.710 (95% CI: 0.631 – 0.788, *P* < 0.001); diagnostic accuracy improved even more when these data were combined with PSA categories (AUC = 0.812, (95% CI: 0.740 – 0.885, *P* < 0.001).

**Conclusions:**

Urinary β2M, PGA3, and MUC3, when analyzed alone or when multiplexed with clinically defined categories of PSA, may be clinically useful in noninvasively resolving the dilemma of effectively discriminating between BPH and localized PCa.

**Electronic supplementary material:**

The online version of this article (doi:10.1186/s12885-015-1284-z) contains supplementary material, which is available to authorized users.

## Background

Prostate cancer (PCa) is the second most frequently diagnosed form of cancer in the world and the sixth most deadly form of male cancer [1] worldwide. In 2013, in the United States alone, it is projected that prostate cancer will account for 28% (238,590) of cancer cases and 10% (29,720) of cancer deaths [2]. Benign prostatic hyperplasia (BPH) is the most common benign disease among men worldwide and its incidence increases with age. PCa and BPH are common diseases of the aging male [3] and share similar symptoms of frequent urination, nocturia, hematuria, dysuria, difficulty starting and maintaining a steady stream of urine, erectial dysfunction, and painful ejaculation [4-8]. An elevated serum prostate-specific antigen (PSA) can be detected with either benign or malignant growth of the prostate. It is the demographic overlap of BPH and prostate cancer, and the lack of discrimination between these two prostate diseases by PSA, that defines the diagnostic dilemma clinicians face when treating prostate disease.

The PSA test has been widely used as a diagnostic, screening, and monitoring tool since it was first approved by the US Food and Drug Administration in 1986 as an aid for the early detection of prostate cancer [9]. However, this test lacks high sensitivity and specificity for PCa and PSA levels are frequently elevated in benign conditions, including BPH [10]. PSA levels do not reliably differentiate between benign and malignant prostate growth. The Prostate Cancer Prevention Trial, conducted on 2950 men, reported that men who had never had a PSA greater than 4.0 ng/mL and who had normal digital rectal exams had a prostate cancer prevalence of 15.2% in this population [11]. Even patients with the lowest PSA levels (up to 0.5 ng/mL) had a prevalence of prostate cancer of 6.6%. While the risk of prostate cancer increases with PSA level, it does not reliably discriminate between benign and malignant disease. For PSA levels between 4.1 to 10.0 ng/mL, the positive predictive value (PPV) for prostate cancer is 25% and, for PSA levels greater than 10 ng/mL, reported PPVs have ranged from 42 to 64% [12,13]. This leads to a situation in which men with indolent disease or BPH continue to be overdiagnosed and unnecessarily biopsied [14]. Side effects of prostate biopsies have been associated with infection, bleeding, urinary difficulty, fever, urinary retention, prostatitis, urosepsis, hematuria, and hematospermia [15]. A variety of permutations of PSA have been utilized to attempt to enhance the diagnostic sensitivity and specificity of PSA screening. However, age-adjusted PSA ranges, PSA velocity, PSA density, and free PSA fraction have all been disappointing in their ability to discriminate between BPH and prostate cancer [16], making it very difficult to differentiate between these two diseases in a clinical setting. In May of 2012, the United States Preventive Services Task Force (USPSTF) announced that PSA screening for prostate cancer demonstrated small potential benefit against a backdrop of potential harms [17], including misdiagnosis.

The early differential diagnosis between BPH and prostate cancer is essential given the fact that both the outcome and the treatment of these two prostatic diseases are distinct [18]. Currently, prostate cancer prognosis is based on age, elevated levels of PSA, and a prostatic digital rectal examination (DRE) often followed by prostate biopsy [19], none of which can distinguish between BPH and prostate cancer [18]. There is, therefore, an urgent need for novel biomarkers that can effectively distinguish between patients with BPH vs. PCa.

The goal of this study was to identify and validate non-invasive urinary biomarkers that distinguish between BPH and localized PCa. With the advantage of being in direct continuity with the prostatic lumen, urine represents a body fluid that is enriched with proteins from PCa cells [19] making it useful source of proteins for biomarker discovery. In addition, urine-based tests are truly noninvasive [20] and more easily accessible than other methods, including blood.

## Methods

### Urine collection and processing

This study was approved, and urine collected, according to the institutional bioethical guidelines of the Institutional Review Board at the Brigham and Women’s Hospital (Boston). Samples were obtained in the Urology Clinic at the Brigham and Women’s Hospital (Boston) before surgical or other therapeutic interventions. All participants gave informed consent. The diagnosis of BPH was made on the basis of clinical parameters which included a normal DRE and either a normal PSA level or a prior negative prostate biopsy if the PSA was elevated. The diagnosis of prostate cancer was made only in those patients who had confirmatory positive biopsies without evidence of metastatic disease. The patients diagnosed with BPH were followed up for 5 years and were declared prostate cancer free. Samples were collected in sterile containers as voided urine and immediately frozen at −20°C and stored as previously reported [21]. Urine was tested for the presence of blood and leukocytes using Multistix 9 strips (Siemens Healthcare Diagnostics Inc., Tarrytown, NY), and samples containing blood or leukocytes were excluded [21]. None of the patients had clinical signs or documentation of prostatitis or UTI.

One hundred seventy-three (173) samples were analyzed in this study, including samples from patients diagnosed with benign prostate hyperplasia (n = 83) and prostate cancer (n = 90). Specimens taken from patients with localized cancers were obtained prior to surgical or other therapeutic intervention. The two groups were not significantly different with respect to race (% Caucasian: BPH 80%, PCa 74% *P* = 0.11, chi-square test). Gleason scores of the prostate adenocarcinomas ranged from 5 to 9, with 85 out of the 90 (94%) prostate adenocarcinomas having Gleason scores of 5–7. We had one patient who was graded as having a Gleason score 5 by the pathologist. This was very early in the series. The prostate cancer group included stages T1-T3 with only one patient diagnosed as a T1a stage on TURP. None of the patients were diagnosed with metastatic cancer at the time of sampling. Mean age was not significantly different between the PCa and BPH and PCa groups (63.3 vs. 66.1 years, *P* = 0.15) as reported in Table 1. Samples were analyzed in a double-blinded manner.

**Table 1:** **Characteristics of study groups**

Characteristics	Number of PCa patients (%)	Number of BPH patients (%)
**Number of patients**	90	83
**Age**, years, mean ± SD	66.1 ± 8.4	63.3 ± 8.7
**Race**		
Caucasian	67 (74.44)	66 (79.5)
Black	10 (11.1)	5 (6.0)
Other	13 (14.44)	12 (14.4)
**Ethnicity**		
African-american	3 (3.3)	4 (4.8)
Hispanic	5 (5.55)	0 (0)
Unknown	57 (63.3)	47 (56.6)
Other	25 (27.7)	32 (38.55)
**Biopsy grade**		
Gleason 5	1 (1.11)	
Gleason 6	47 (52.22)	
Gleason 7	37 (41.11)	
Gleason ≥ 8	5 (5.55)	
**Biopsy stage**		
T1a	1 (1.11)	
T1c	7 (7.77)	
T2a	24 (26.66)	
T2b	3 (3.33)	
T2c	43 (47.77)	
T3a	7 (7.77)	
T3b	5 (5.55)	

### LC/LC/MS/MS identification of differentially expressed proteins by isobaric tagging with iTRAQ (*isobaric tags for relative and absolute quantitation)*

iTRAQ is a state-of-the-art quantitative mass spectrometry approach to identify and quantify components of the proteome present in biological samples. Proteins were obtained from human urine by organic precipitation with methanol following our previously published method [22]. Protein profiling was performed using the 8-plex iTRAQ (AB Sciex, Foster City, CA) labeling protocol and standard MudPIT methodology coupled with the 4800 MALDI TOF/TOF Plus instrument to perform the mass spectrometry as previously described [22]. Protein Pilot 2.0.1 software with the Paragon algorithm [23] was used for peptide and protein identification and relative quantitation based on the iTRAQ labels (Additional file 1).

### Immunoblot analyses

All 173 urine samples (BPH = 83, PCa = 90) were individually concentrated using an UltraFree-4 centrifugal filter device with a molecular weight cut off of 5 kDa (Millipore, Bedford, MA, USA) as previously reported [21,24]. Protein expression was detected by immunoblot analyses using monospecific primary antibodies: apoD, β2M, pepsin A, uromodulin, ZAG, (Santa Cruz Biotechnology, Santa Cruz, CA, USA) and MUC3 (Thermo Fisher Scientific Inc, Rockford, IL, USA). Proteins were evaluated via quantitative densitometry and expressed in densitometric units (DU) (Additional file 1).

### Pathways and interactive network systems biology analysis

Ingenuity Pathway Analysis (IPA 7.0) (http//www.ingenuity.com) was used to identify key interaction networks and pathways significantly enriched in BPH and PCa urine samples. Based on the differentially expressed proteins, we built a network composed of interactive proteins using the network building and growing utility in the Ingenuity Pathway Analysis (IPA) tool. Enriched pathways within this hierarchical network were ranked using the ratio of affected proteins and Fisher’s exact test. To identify the key regulatory molecules within this integrated network, we used the density of maximum neighborhood component (DMNC) algorithm [25].

### Statistical analysis

Univariate analysis included a comparison of median levels of the six proteins and PSA between PCa and BPH patients by the nonparametric Mann–Whitney *U*-test since these variables showed some skewness and lack of normality as tested by the Kolmogorov-Smirnov goodness-of-fit statistic. Receiver operating characteristic (ROC) curve analysis was applied to determine the area under the curve (AUC) as a measure of predictive accuracy and the Youden J-index was used to identify the optimal cutoff value for each biomarker [26]. Multivariate logistic regression analysis using backward selection with the likelihood ratio test to assess significance was applied to identify the independent predictive biomarkers of PCa and derive the probability of PCa based on combinations of these biomarkers (using the optimal cutoff value in densitometric units) and stratified by PSA level based on three clinical categories (0–4, 4.1-10, >10 ng/ml). AUC was also calculated for combinations of biomarkers with 95% confidence intervals to determine whether improved prediction was achieved by combining significant biomarkers together using multivariate modeling with the *c* index used to quantify the combined predictive accuracy [27]. ROC curves were compared by the DeLong test [28]. Two-tailed values of *P* < 0.05 were considered statistically significant. Statistical analysis was performed using IBM SPSS Statistics (version 21.0, IBM, Armonk, NY). This study was conducted and its results reported in accordance with the REMARK guidelines [29].

## Results

**Figure 1:**
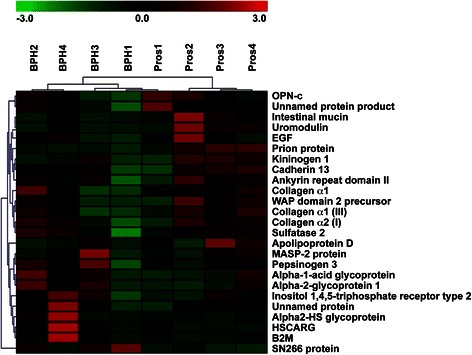
Urinary proteins significantly differentially expressed between BPH vs. PCa identified by iTRAQ. The relative level of protein expression is shown with a pseudo color scale (−3 to 3), with red denoting up-regulation and green denoting down-regulation. The columns represent samples and the rows represent the proteins.

**Figure 2:**
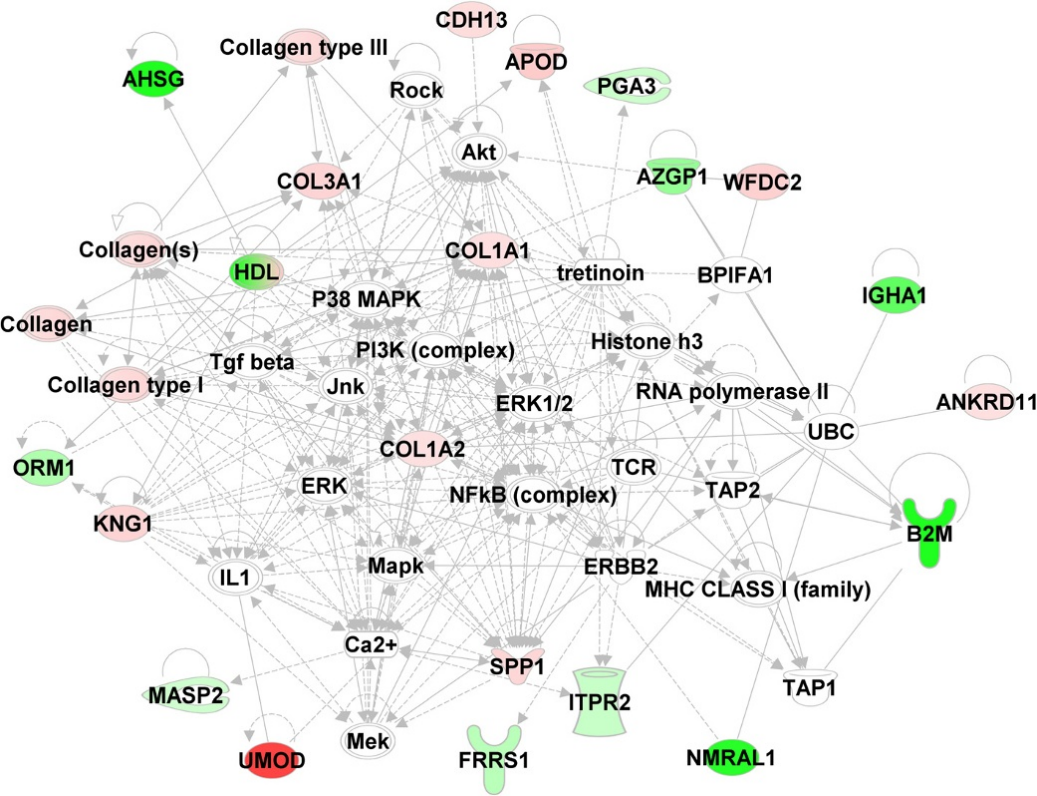
Interactive network of the proteins that are differentially expressed in prostate cancer as compared to BPH. The network node and edges represent proteins and their interactions respectively. The intensity of the node color indicates the degree of up-regulation (red) or down-regulation (green), while white nodes indicate non-modified proteins that may be affected by post-translational modification. All networks shown were significantly affected in prostate cancer, with a score >15. The network analysis identified many focus hubs (e.g. NFκB, ERK1/2, Collagen, TGFβ, PI3K, p38 MAPK) with high degree of interactions.

We utilized iTRAQ LC/LC/MS/MS as our technique of choice to sensitively and accurately identify the urinary proteome of men with BPH vs. PCa. This approach identified 25 proteins that were differentially expressed, both at a significant level and with high confidence, in urines from men from each of the two groups of interest (PCa and BPH) (Figure 1). We then performed functional enrichment analysis and pathways enrichment analysis of these proteins using Ingenuity Pathway Analysis (IPA) tools to determine differentially expressed pathways and functions in PCa as compared to BPH. These proteins represent a number of different functional categories including cell assembly and organization, cell signaling, cell morphology, carbohydrate metabolism, cellular growth and proliferation, lipid metabolism, androgen and estrogen metabolism, and DNA replication, recombination, and repair, among others (Additional file 2: Figure S1). Additionally, network analysis identified differences in many focus hubs (e.g. NFκB, ERK1/2, Collagen, TGFβ, PI3K, and p38 MAPK) with a high degree of interactivity (Figure 2).

**Figure 3:**
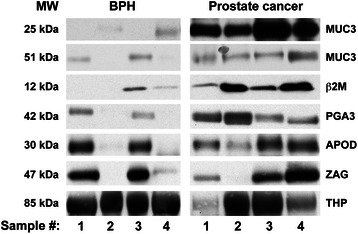
Immunoblot analyses of urine samples from BPH and prostate cancer patients. Representative urine samples were separated on 4-12% Bis-Tris gels under reducing conditions and were subsequently subjected to western blot analysis using the appropriate antibody for the following iTRAQ-identified proteins: mucin 3 fragment (MUC3 25 kDa), mucin 3 fragment (MUC3 51 kDa), β-2-microglobulin (β2M), pepsinogen 3 (PGA3), apoliprotein D (ApoD), alpha-2-glycoprotein 1, zinc (ZAG) and uromodulin (THP). One hundred seventy-three (173) urine samples from patients diagnosed with benign disease (N = 83) and tumor disease (N = 90) were analyzed.

25 proteins were differentially expressed in the urine of patients diagnosed with BPH vs. PCa as originally identified by iTRAQ (Figure 1). Given that enzyme-linked immunosorbent assays (ELISA) for these proteins were not available, nine proteins were tested based on the availability of antibodies. Of these nine, six proteins were validated: β-2-microglobulin (β2M), pepsinogen 3, group 1 (PGA3), intestinal mucin (MUC3), apolipoprotein D (APOD), alpha-2-glycoprotein 1, zinc (ZAG), and uromodulin (THP) (Figure 3).

**Figure 4:**
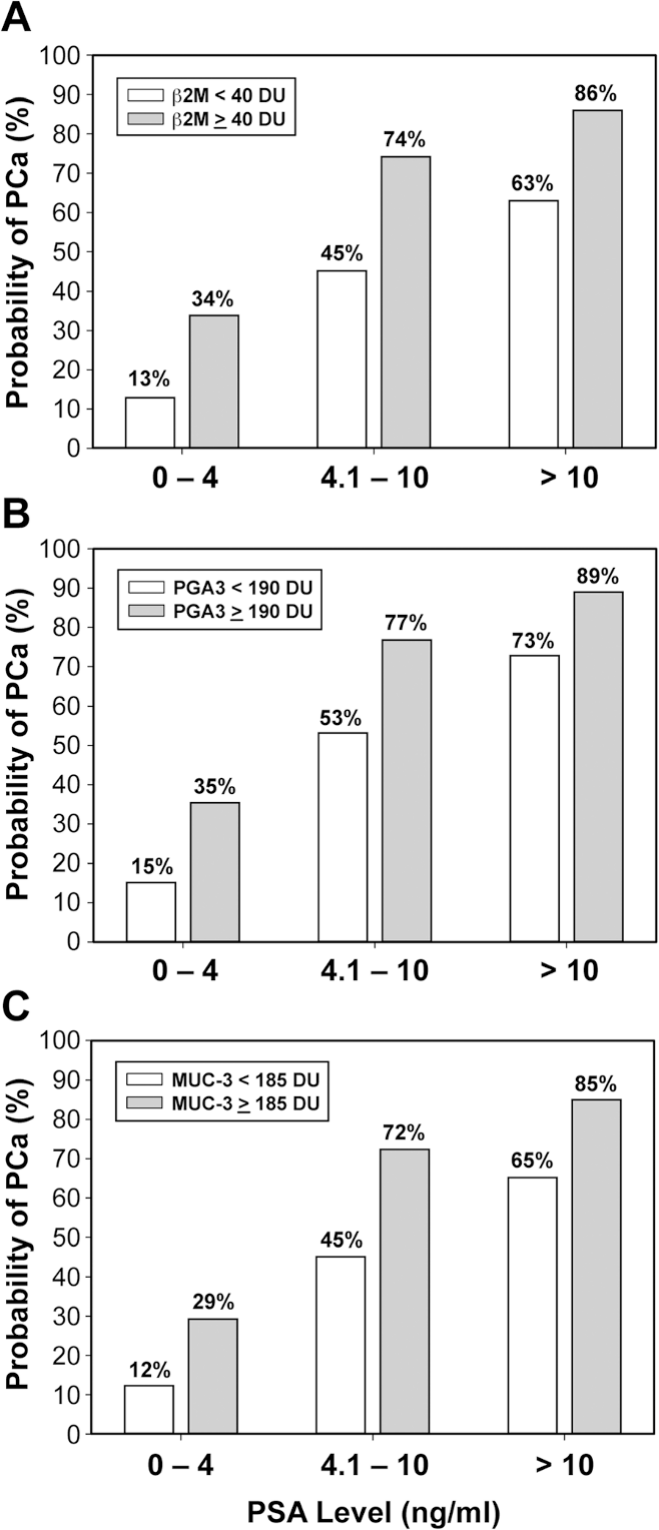
Probability of PCa according to urinary biomarkers stratified by PSA level: **A**) Probability of PCa according to β2M stratified by PSA level, β2M ≥ 40 DU (*P* <0.001) **B**) Probability of PCa according to β2M stratified by PSA level, PGA3 ≥ 190 DU (*P* = 0.008) **B**) Probability of PCa according to PGA3 stratified by PSA level **C**) Probability of PCa according to MUC3 stratified by PSA level, MUC3 ≥ 185 DU (*P* = 0.009).

**Figure 5:**
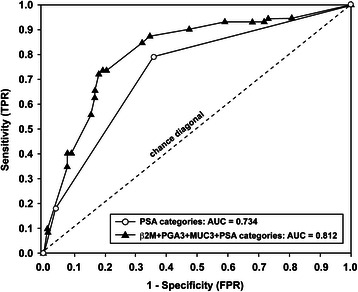
Receiver operating characteristic curves for combined urinary biomarkers in differentiating BPH patients from PCa patients. White circles represent the ROC curve (AUC = 0.734) for three clinically relevant PSA categories (0–4, 4.1-10, >10 ng/mL). Black triangles signify the ROC curve based on the combination of three urinary biomarkers with PSA categories and demonstrate the highest diagnostic accuracy (AUC = 0.812), representing significant improvement (*P* = 0.004).

Univariate analysis was performed to compare BPH and PCa groups with respect to age and each of the six validated urinary biomarkers (Table 2). These comparisons, based on continuous data of the six validated proteins, revealed significant elevations in β2M (*P* < 0.001), PGA3 (*P* = 0.006), and MUC3 25 kDa (*P* = 0.018). We then identified the optimal cut-off values using ROC analysis with the Youden index for each of three significant urinary biomarkers, and the analysis indicated ≥ 40 DU for β2M, ≥ 190 DU for PGA3, and ≥ 185 DU for MUC3. Multivariate logistic regression modeling was then conducted using the chosen cut-off values for each of the three significant biomarkers in the univariate analysis as well as for PSA (using three clinically defined categories: 0–4 ng/mL, 4.1-10 ng/mL, >10 ng/mL). The probability of PCa was determined using multivariate logistic regression modeling according to each of three urinary biomarkers (β2M, PGA3, MUC3) as well as PSA in the predictive model. Having determined the optimal cut-off value for each biomarker based on the Youden J-index in receiver operating characteristic (ROC) curve analysis, we chose to use the binary predictors (i.e., above and below each cut-off) stratified according to clinically relevant categories of PSA. The probabilities shown in each panel of Figure 4 are based on two levels of the biomarker for each of three PSA categories (0–4, 4.1-10, >10 ng/mL). The multivariate modeling strategy uses the Newton–Raphson algorithm in maximum likelihood estimation (MLE) to derive the probability of PCa based on combinations of the biomarkers within each PSA category. Figure 4 illustrates that the estimated probability of PCa within each PSA category is significantly higher in patients who are above the cut-off value for the biomarker and that the probability of PCa is elevated with increasing PSA. ROC analyses revealed that if the levels of β2M are less than 40 DU, the PGA3 levels are less than 195 DU and the MUC3 levels are less than 185 DU, the predictive accuracy is improved to 45%, 53%, and 45%, respectively. However, when the DU levels are equal to, or higher than, 40 DU of β2M, 195 DU of PGA3 and 185 DU of MUC3, the diagnostic accuracy is significantly improved to 74%, 77% and 72%, respectively (Figure 4). In addition, the ROC clearly shows a steeper curve for the three urinary biomarkers (MUC3, PGA3, β2M), as well as for the three biomarkers and the PSA categories (Figure 5).

**Table 2:** **Comparison of Age and Urinary Proteins Between PCa and BPH Cohorts**

Variable	PCa	BPH		
	**(N = 90)**	**(N = 83)**	**AUC**	***P*** **value**
Age, years, mean	63.3 ± 8.7	66.1 ± 8.4	–	0.15
β2M	143.4 (44.5-289.8)	30.3 (4.2-194.6)	0.658	<0.001*
PGA3	198 (32–329)	106 (8–263)	0.623	0.006*
MUC3 25 kDa	421 (239–490)	322 (93–465)	0.605	0.018*
MUC3 51 kDa	33.0 (6.3-104.9)	18.7 (3.2-68.4)	0.583	0.06
APOD	381 (134–512)	255 (35–486)	0.568	0.14
THP	267 (122–368)	232 (89–377)	0.547	0.30
ZAG	384 (144–529)	351 (89–523)	0.522	0.64

Biomarker data are median (interquartile range) of densitometric units (DU). AUC = area under the curve. * Statistically significant.

To determine the predictive accuracy of each of the significant independent multivariate biomarkers based on the optimal cutoff values and PSA based on the three categories, we used ROC analysis to assess the AUC for single biomarkers and the combination of three with and without PSA (Table 3). Single biomarkers had AUC values ranging from 0.618 for MUC3 25 kDa (*P* = 0.009) to 0.668 for β2M (*P* < 0.001); the combination of β2M, PGA3 and MUC3 25 kDa increased the AUC to 0.710 (95% CI: 0.631 – 0.788, *P* < 0.001). Predictive accuracy was 0.734 based on PSA categories alone and significantly increased to 0.812 for the three biomarkers combined with PSA categories (*P* = 0.004, Delong test for comparing ROC curves). False positive (FPR) and false negative rates (FNR) are highly relevant in clinical practice and we have evaluated the FPR and FNR for each of the three significant multivariate predictive biomarkers in differentiating between BPH and PCa. It is clear that compared to each of the three urinary biomarkers alone, our combined panel of three biomarkers provide much lower FPRs and FNRs based on all patients in the study population. The primary objective of this study was to identify the best set of urinary biomarkers to improve diagnostic accuracy in differentiating between BPH and PCa; the misclassification rates shown above underscore the value of a panel of biomarkers rather than any one biomarker in isolation. The combination of three biomarkers together (β2M, PGA3, MUC3) shows an FPR of 30% in conjunction with a very low FNR range of 0% to 8%, making this combination potentially useful in a clinical setting.

**Table 3:** Diagnostic accuracy of biomarkers in predicting PCa based on optimal cutoff values from ROC analysis*

Biomarker	AUC	95% CI	*P*value
β2M ≥ 40 DU	0.668	0.628 – 0.748	<0.001*
PGA3 ≥ 190 DU	0.625	0.547 – 0.710	0.008*
MUC3 ≥ 185 DU	0.618	0.532 – 0.700	0.009*
PSA categories, ng/mL (0–4.0, 4.1-10, >10)	0.734	0.653 – 0.814	0.007*
β2M + PGA3 + MUC3	0.710	0.631 – 0.788	<0.001*
β2M + PGA3 + MUC3 + PSA categories	0.812	0.740 – 0.885	<0.001*

AUC = area under the curve; CI = confidence interval, DU = densitometric unit. * Determined by the Youden index.

## Discussion

This study was designed to evaluate new markers in a patient population that would undergo screening in common clinical practice. The U.S. Preventive Task Force has rejected the utility of PSA screening for prostate cancer and this study was designed to determine if new urinary markers would be more informative in discriminating between BPH and prostate cancer which is the problem facing clinicians. Neither PSA nor these urinary markers are intended to discriminate between indolent versus aggressive prostate cancer, they are not intended to be utilized to identify “normal” men who have neither BPH nor prostate cancer. The clinical challenge is differentiating BPH from prostate cancer. Although transition zone cancers can account for 10-20% of prostate cancers, they are not normally diagnosed with typical initial screening strategies. They are usually considered when one or more initial sets of routine transrectal biopsies have been negative for prostate cancer and clinical suspicion persists, and such patients often undergo MRI imaging and more extensive biopsy regimens. These patients were not included in our routine screening population.

In recent years, urinary biomarkers have emerged as an attractive option for the noninvasive detection of PCa [21,30-32]. Given the complexity of this disease, it is now widely appreciated that a single marker may not necessarily reflect the multifactorial nature of BPH or PCa [30]. A panel, rather than any individual biomarker, will have a higher likelihood to more accurately distinguish between BPH and localized PCa in conjunction with clinico-pathological parameters. This panel of three newly identified biomarkers β2M, PGA3, and MUC3 effectively discriminated BPH from localized PCa.

The first protein found to be significantly elevated in urine of PCa patients was mucin 3 (MUC3), a member of the membrane-associated mucins, which may be shed from the cell surface via activation of membrane-associated metalloproteinases [33-35]. Previous studies reported a correlation between elevated MUC3 expression and esophageal [36], gastric [37], breast [38], and colon cancers [39]. We found that MUC3 was able to differentiate between BPH and localized PCa. In addition, this ability of MUC3 to discriminate between BPH and localized PCa was strengthened when MUC3 was multiplexed with clinically-defined categories of PSA, making it a prospective biomarker for differentiating BPH from localized PCa.

We also found Pepsinogen 3, group 1 (PGA3) to be elevated in the urine of PCa patients but not in BPH. PGA3 is synthesized and secreted by the gastric chief cells of the human stomach before being converted into the proteolytic enzyme pepsin A, an upstream step in the digestive process [40]. Low levels of PGA in serum [41], as well as decreased or lost expression of PGA in gastric tissue and cancer cell lines, were previously reported [42]. In contrast, a recent study demonstrated increased mRNA levels of PGA in seven colorectal cancer cell lines [43]. Interestingly, our study is the first to report that PGA3 can be used to effectively distinguish between patients with BPH or with localized PCa.

Lastly, β2M, a component of the major histocompatibility complex class I (MHC I), was the third protein identified via iTRAQ and validated by immunoblot analysis. Increased expression of β2M has been previously associated with breast [44], renal [45], lung [46], colon [47], and hematologic malignancies [48]. β2M levels were also significantly elevated in urine [49] and in serum [50] of prostate cancer patients when compared to healthy subjects. Ours is the first study demonstrating that β2M effectively discriminates between BPH and localized of PCa.

## Conclusions

This comprehensive iTRAQ LC/LC/MS/MS analysis, followed by extensive validation of the candidate urinary biomarkers, revealed that β2M, PGA3, and MUC3 can sensitively and specifically differentiate between patients with BPH and localized prostate cancer. When these markers are multiplexed, their accuracy in differentiating between BPH and localized PCa is further increased. Importantly, this small panel of biomarkers, when multiplexed with clinically defined categories of PSA, effectively distinguishes BPH from localized PCa with high sensitivity and specificity. In summary, noninvasive urine tests utilizing β2M, PGA3 and MUC3, in conjunction with clinically defined categories of PSA, have the potential to significantly enhance the ability to discriminate between BPH and localized PCa in the clinical setting.

## Additional files

Additional file 1:
**Supplemental methods.**

Additional file 2: Figure S1. Supplemental figure.

## Abbreviations

BPH: Benign prostate hyperplasia
PCa: Prostate cancer
PSA: Prostate specific antigen
MUC3: Mucin 3
PGA3: Pepsinogen 3, group 1
β2M: β-2-microglobulin
