# Supplementary material for: Novel non-invasive biomarkers that distinguish between benign prostate hyperplasia and prostate cancer
Source: BMC Cancer. 2015 Apr 11;15:259. doi: 10.1186/s12885-015-1284-z (PMC4433087; doi:10.1186/s12885-015-1284-z)
Supplement: Additional file 1: — Supplemental methods. [file 12885_2015_1284_MOESM1_ESM.pdf]

Supplemental Figure Legends

Supp. Figure 1. Pathways and functional enrichment analysis of proteins differentially expressed in prostate cancer as compared to BPH: A) Functional enrichment analysis and B) Pathways Enrichment analysis. For Figure 1A and 1B each bar represents a significantly enriched pathway or function as determined by the multiple tests corrected Fisher's Exact Test P value. The P value is depicted as –log10 (B-H P value) on primary X-axis. The analysis for canonical pathways and functions was performed using Ingenuity Pathway Analysis.

Supplementary Methods

Chemicals

Methanol, ethanol, and 10% formalin were purchased from Thermo Fisher Scientific Inc. (Waltham, MA, USA). Ethanol, EDTA, 2-mercaptoethanol, bovine serum albumin (BSA), and Triton x-100 were purchased from Sigma (St. Louis, MO, USA). Tween 20 and Bradford protein reagent were purchased from Bio-Rad (Hercules, CA, USA).

Immunoblot analyses

Protein concentration of urine samples was determined by the Bradford method (Bio-Rad, Hercules, CA, USA). Equal amounts of proteins from urine (30 μg/lane) were separated on NuPAGE 4-12% Bis-Tris gels (Invitrogen, Carlsbad, CA, USA) and transferred to PVDF membranes (Millipore, Bedford, MA, USA). Protein expression was determined by appropriate primary antibodies: apoD, β-2M, pepsin A, uromodulin, ZAG, (Santa Cruz Biotechnology, Santa Cruz, CA, USA) and MUC3 (Thermo Fisher Scientific Inc, Rockford, IL, USA) diluted to 1:1000 in 5% BSA blocking solution. Secondary anti-mouse and anti-rabbit antibodies 1:2000 (Thermo Fisher Scientific Inc, Rockford, IL, USA) were diluted in 5% BSA. The expression of each protein was detected by the ECL Western Lightning (PerkinElmer Life and Analytical Sciences, Inc., Waltham, MA) as previously reported [1,2] and bands of interest corresponding to target proteins (apoD, β2M, pepsin A, uromodulin, ZAG, and MUC3) were evaluated via quantitative densitometry (Un-Scan-It, Silk Scientific Inc., Orem, UT).

LC/LC/MS/MS identification of differentially expressed proteins by isobaric tagging with iTRAQ

Briefly, ice-cold 100% methanol was added to ice-cold urine to a final concentration of 75%. After mixing by inversion several times the tubes were stored overnight at -20 °C. Precipitated proteins were collected by centrifugation (16,000 x g, 15 min at room temperature). Precipitated proteins were washed three times with ice-cold 100% methanol and five-minute spins were employed to recover the protein after each wash. Collected proteins from each sample were then briefly air-dried in a fume hood and resuspended in milliQ water to an approximate concentration of 1.0 mg/ml. Protein concentration was determined using the BioRad Protein Assay (BioRad, Hercules, CA, USA). A standard curve was generated using bovine serum albumin (BSA) and all assays were performed in triplicate with the mean value used for final calculations. All samples were analyzed by Coomassie stained SDS-PAGE (10% Tris-HCl Ready Gel, BioRad, Hercules, CA, USA) to gauge the relative distribution of proteins in each sample versus intact albumin. Ten micrograms of protein was loaded per well and the gel assay confirmed stained protein in each sample.

Equal amounts of total protein from the human urine samples were used in the 8-plex-iTRAQ relative quantitation protocol following the manufacturer's recommendations (AB Sciex, Foster City, CA, USA). Four urine samples from BPH patients and four samples from prostate cancer patients were used in one 8-plex-iTRAQ experiment. One hundred micrograms from each individual urine was dried in a centrifugal speed vacuum set-up. Each dried sample was then resuspended in 20 μl of dissolution buffer (0.5 M

triethylammonium bicarbonate (TEAB), pH 8.5) containing 0·1% SDS. Proteins were reduced by adding 2 μl of 50 mM tris-(2-carboxymethyl) phosphine (TCEP) and incubated at 55 °C for one hour. Reduced disulfide bonds were then blocked by adding 1 μl of 200 mM methyl methanesulfonate (MMTS) and incubating at room temperature for 10 minutes. Proteins were digested into peptides using overnight trypsin digestion at 37 °C (added in ratio of 1:10). All peptides were then reacted with the 8-plex iTRAQ labels. After confirmation of addition of each label to each digested sample all eight samples were then pooled into one tube. Peptides were identified using the MudPIT methodology. First, peptides were separated on a strong cation exchange (SCX) column (POROS HS/20, 4.6 mm X 100 mm (Applied Biosystems, Foster City, CA, USA) attached to an Agilent 1100/1200 HPLC. Buffer A was 10 mM KH_2PO_4, 25% (vol/vol) acetonitrile, pH 2.8 and Buffer B was Buffer A plus 1 M KCl. A total of ninety-six fractions were collected. Based on the chromatogram at UV 214 the eluted fractions were pooled back into thirty tubes that spanned the range of resolved peptides.

In the second dimension separation, each pooled SCX fraction was then run over a reverse phase column (Acclaim PepMap100 C18, 5 μm, 100A, 500 μm ID X 5 mm, Dionex) using microflow and then resolved with nanoflow LC on an Ultimate Plus system (LC Packings/Dionex) over a 15 cm C18 column (Acclaim PepMap100, 3 μm, 75 μm ID x 15 cm, Dionex) and then printed to 4800 MALDI target plates with an attached printing robot (Probot, Dionex). The MALDI matrix CHCA (Sigma, St. Louis, MO, USA) was made as a 5.0 mg/ml stock solution and mixed in automatically by the Probot just prior to printing the samples on to the target plates. Peptides were identified by MALDI-TOF/TOF mass spectrometry on a 4800 Plus instrument (AB Sciex, Foster City,

CA, USA). Protein Pilot 2.0.1 software was used for peptide and protein identification and relative quantitation based on the iTRAQ labels. Protein Pilot used the Paragon algorithm [3]. The latest available versions of three human protein databases were separately searched using Protein Pilot 2.0.1; SwissProt (non-redundant, 18 053 proteins), TrEMBL (redundant, 55 942 proteins) and NCBI (redundant, 176 140 proteins).

Supplementary Methods References

1. Moses MA, Wiederschain D, Loughlin KR et al: **Increased incidence of matrix metalloproteinases in urine of cancer patients.** Cancer Res 1998, **58:**1395-1399.
2. Roy R, Louis G, Loughlin KR et al: **Tumor-specific urinary matrix metalloproteinase fingerprinting: identification of high molecular weight urinary matrix metalloproteinase species.** Clin Cancer Res 2008, **14:**6610-6617.
3. Shilov IV, Seymour SL, Patel AA et al: **The Paragon Algorithm, a next generation search engine that uses sequence temperature values and feature probabilities to identify peptides from tandem mass spectra.** Mol Cell Proteomics 2007, **6:**1638-1655.
