# Supplementary figures and images for: Novel non-invasive biomarkers that distinguish between benign prostate hyperplasia and prostate cancer
Source: BMC Cancer. 2015 Apr 11;15:259. doi: 10.1186/s12885-015-1284-z (PMC4433087; doi:10.1186/s12885-015-1284-z)

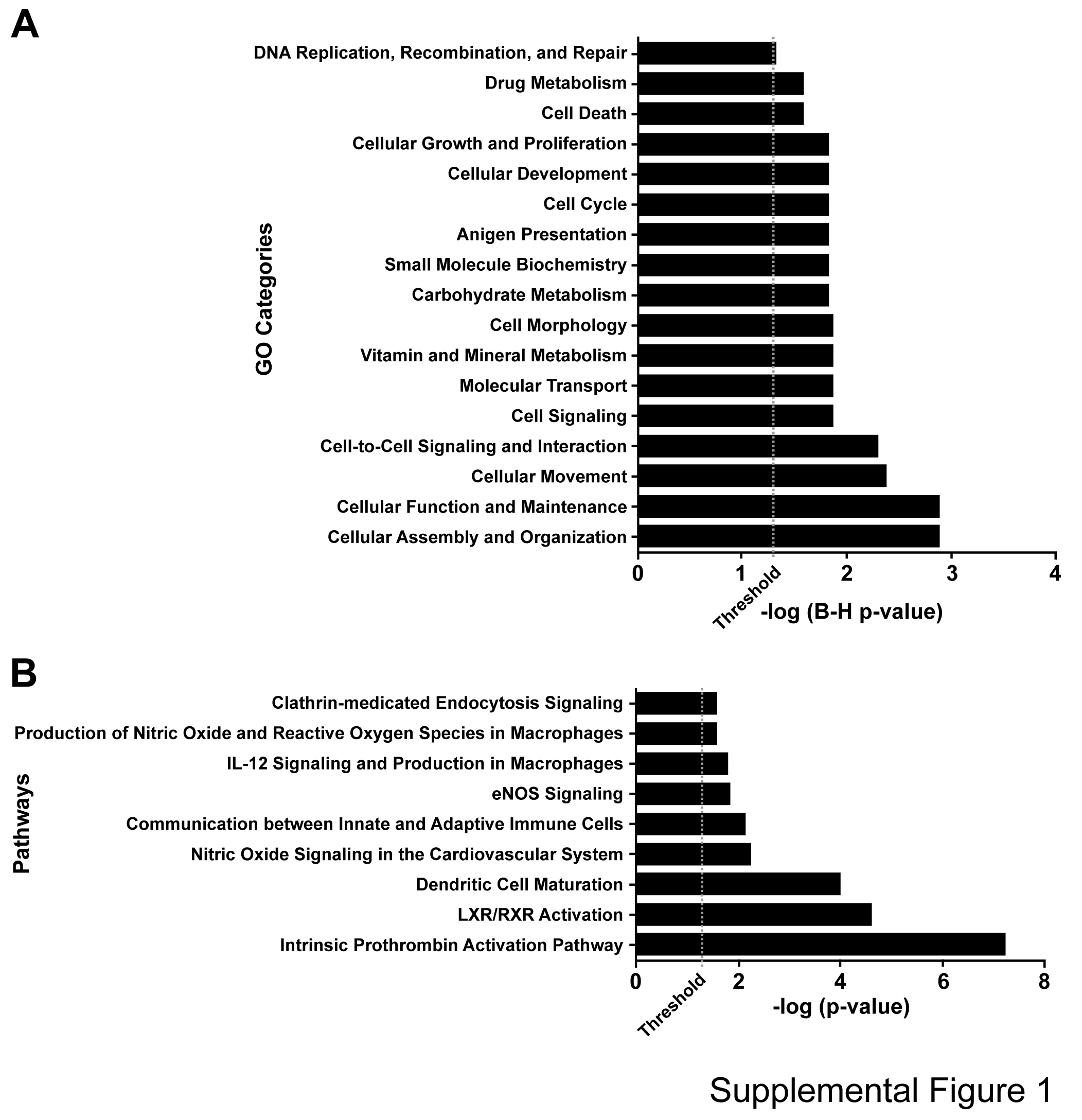

Supplement: Additional file 2: Figure S1. — Supplemental figure. [file 12885_2015_1284_MOESM2_ESM.tiff]
